# Comparison of the upper airway morphology between Dutch and Chinese adults with obstructive sleep apnea

**DOI:** 10.1007/s11325-023-02834-z

**Published:** 2023-04-24

**Authors:** Xiaoxin Shi, Hui Chen, Frank Lobbezoo, Jan de Lange, Paul van der Stelt, Erwin Berkhout, Jing Guo, Shaohua Ge, Guoju Li, Yanzhong Li, Ghizlane Aarab

**Affiliations:** 1 Department of Orofacial Pain and Dysfunction, Academic Centre for Dentistry Amsterdam (ACTA), University of Amsterdam and Vrije Universiteit Amsterdam, Amsterdam, The Netherlands; 2 Department of Oral Radiology, Academic Centre for Dentistry Amsterdam (ACTA), University of Amsterdam and Vrije Universiteit Amsterdam, Amsterdam, The Netherlands; 3 Department of Oral and Maxillofacial Surgery, Amsterdam University Medical Centers/Academic Centre for Dentistry Amsterdam (ACTA), University of Amsterdam, Amsterdam, The Netherlands; 4 Department of Orthodontics and Periodontics and Oral Radiology, School and Hospital of Stomatology, Cheeloo College of Medicine, Shandong University & Shandong Key Laboratory of Oral Tissue Regeneration & Shandong Engineering Laboratory for Dental Materials and Oral Tissue Regeneration, No. 44-1 Wenhua Road west, Jinan, 250012 China; 5 Department of Otorhinolaryngology, Qilu Hospital of Shandong University, NHC Key Laboratory of Otorhinolaryngology, Jinan, China

**Keywords:** Obstructive sleep apnea, Races, Upper airway morphology, Anatomical balance, Cone beam computed tomography

## Abstract

**Purpose:**

The pathogenesis of obstructive sleep apnea (OSA) is complex and may vary between different races. It has been suggested that the anatomical balance between skeletal tissues and soft tissues around the upper airway is a key pathophysiologic factor of OSA. Therefore, the aim of this study was to compare the anatomical balance of the upper airway between Dutch and Chinese patients with OSA based on cone beam computed tomography (CBCT) images.

**Methods:**

This was a cross-sectional study performed in two centers and included Dutch and Chinese adults with OSA. CBCT scans in the supine position were obtained for both Dutch and Chinese OSA groups. The primary outcome variable was the anatomical balance of the upper airway, defined as the ratio of the tongue area and the maxillomandibular enclosure area.

**Results:**

A total of 28 Dutch adults (mean age ± SD of 46.6 ± 14.1 years, body mass index [BMI] of 26.8 ± 3.5 kg/m^2^, and apnea-hypopnea index [AHI] of 15.7 ± 7.1 events/h) and 24 Chinese adults (age 41.0 ± 12.4 years, BMI 26.5 ± 3.3 kg/m^2^, and AHI 16.5 ± 7.8 events/h). There were no significant differences in AHI, age, BMI, and sex between the two groups (*P* = 0.14–0.76). The Dutch group had a significantly larger tongue area and tongue length compared to the Chinese group (*P* = 0.01 and *P* < 0.01). On the other hand, the Chinese group had a smaller maxilla length compared to the Dutch group (*P* < 0.01). However, the anatomical balance of the upper airway of both groups was not significantly different (*P* = 0.16).

**Conclusion:**

Within the limitations of this study, no significant difference was found in the anatomical balance of the upper airway between Dutch and Chinese patients with mild to moderate OSA.

**Trial registration:**

The present study was registered at the ClinicalTrials.gov identifier NCT03463785.

## Introduction

Obstructive sleep apnea (OSA) is characterized by recurrent complete and/or partial obstructions of the upper airway, often resulting in arousals from sleep and oxygen desaturations [1]. Excessive daytime sleepiness, lack of concentration, and fatigue are examples of frequent complaints of patients with OSA [2]. The pathogenesis of OSA involves a complex interaction of anatomical and non-anatomical factors, among which a narrowed upper airway plays a key role [3]. It has been suggested that the pathogenesis of OSA may vary between different races; however, the exact difference is still unclear [4, 5].

Both restricted skeletal structures and enlarged soft tissues can lead to a narrowed upper airway [6, 7]. Specifically, the anatomical balance of the upper airway, defined as the ratio of the tongue size and the maxillomandibular enclosure size, is a key determinant of upper airway morphology [8]. Studies have indicated that, when the OSA severity is similar, Caucasian adults with OSA are more overweight (e.g., greater body mass index (BMI) and neck circumference), whereas Asian adults with OSA show more severe skeletal restrictions (e.g., a smaller maxilla and mandible, and retrognathia) [9-12]. These findings indicate that the anatomical balance of the upper airway may be similar between the two races.

Only a few studies have performed a direct inter-race comparison of the anatomical balance. However, their results are inclusive. A study of Schorr et al. [11] has suggested that Caucasians with OSA had a greater anatomical imbalance as compared with the Japanese-Brazilians with OSA. On the other hand, the study of Lee et al. [10] has suggested a similar anatomical balance between Caucasian and Chinese patients with OSA. The different results could be due to different races being compared. Besides, their results may be biased by using an inaccurate definition of the anatomical balance. Since the anatomical balance is involved in the pathogenesis of OSA [8, 13], and is also an important predictor of treatment outcome of OSA [14], a better understanding of the role of anatomical balance between different races is necessary. This may help to improve OSA recognition and result in a more targeted therapy for specific racial groups.

According to the current evidence, we hypothesized that the anatomical balance of the upper airway (i.e., the ratio of the tongue area and the maxillomandibular enclosure area) is similar between Dutch and Chinese patients with OSA. To investigate upper airway morphology, cone beam computed tomography (CBCT) has been suggested to be a reliable technique [15, 16] with lower radiation dose and costs compared to traditional CT [17, 18]. Therefore, the aim of this study was to compare the anatomical balance of the upper airway between Dutch and Chinese patients based on CBCT images.

## Material and methods

### Overview

This was cross-sectional study in which participants were recruited from both the Amsterdam University Medical Center (AUMC) in the Netherlands and the Qilu Hospital of Shandong University in China. Dutch participants were recruited from a randomized controlled trial (RCT) (ClinicalTrials.gov identifier: NCT02724865), which was designed to compare the treatment effects of two different types of mandibular advancement device (MAD) in patients with mild and moderate OSA. Chinese participants were recruited prospectively for this study.

The protocol for recruiting the patients from the Netherlands was approved by the Medical Ethics Committee of the AUMC with reference number NL44085.018.13/COSH. The protocol of recruiting patients from China was approved by the Medical Ethics Committee of the Dental School of Shandong University with reference number NO.GR201814.

Written informed consent was obtained from both Dutch and Chinese patients. The present study was registered at the ClinicalTrials.gov identifier NCT03463785.

### Recruitment

Patients that fit the following inclusion/exclusion criteria were recruited in both the Netherlands and China. The inclusion criteria were as follows: (1) age ≥ 18 years; (2) able to speak, read, and write either Dutch or Chinese; (3) able to follow up; (4) diagnosed with symptomatic mild or moderate OSA (5 ≤ apnea-hypopnea index (AHI) < 30); and (5) expected to be able to maintain their current lifestyle (sports, medicine, diet, etc.).

The exclusion criteria were as follows: (1) medication use related to sleeping disorders; (2) evidence of respiratory and/or sleep disorders other than OSA (e.g., central sleep apnea syndrome); (3) systemic disorders (based on medical history and examination, e.g., rheumatoid arthritis); (4) medical history of known daytime fatigue or severe sleep disturbance (e.g., insomnia, PLMS, narcolepsy); (5) known medical history of mental retardation, memory impairment, or psychiatric disorders; (6) reversible morphological upper airway abnormalities (viz., indication for upper airway surgery); (7) syndromes with craniofacial abnormalities (e.g., Pierre Robin sequence and Down syndrome); and (8) inability to provide informed consent. As the Dutch OSA group was recruited from an RCT study, there were two extra exclusion criteria for this group: (1). untreated periodontal problems/toothache/lack of retention possibilities for an MAD, and (2). concomitant use of other modalities to treat OSA.

### Polysomnography (PSG)

All Dutch participants included in this study underwent an overnight PSG recording (Embla A10, Broomfield, CO, USA) at one of the four participating sleep centers (Onze Lieve Vrouwe Gasthuis Ziekenhuis (OLVG), Nederlands Slaap Instituut, Medisch Centrum Jan van Goyen, and Amsterdam Medical Center) in Amsterdam for the diagnosis of OSA. All Chinese participants included in this study underwent an overnight PSG recording (Alice 6, Phillips Respironics, USA) at the Qilu Hospital in Jinan for the diagnosis of OSA.

PSG included the following variables: electroencephalogram, electrooculogram, leg and chin electromyograms, electrocardiogram, pulse oximetry, body position, neck microphone, nasal cannula pressure transducer, and inductive plethysmography by means of thoracic and abdominal bands. The PSG recordings were manually scored in a standard manner for both Dutch and Chinese OSA groups [19]. Apnea was defined as a cessation of airflow of ≥ 90% for at least 10 s. Hypopnea was defined as a decrease in airflow of more than 30% for at least 10 s, accompanied by either ≥ 3% oxygen desaturation or an arousal [19]. The apnea-hypopnea index (AHI) was defined as the number of apneas and hypopneas per hour of sleep.

### Cone beam computed tomography (CBCT)

The CBCT datasets of Dutch and Chinese patients were obtained using identical NewTom 5G CBCT systems (QR systems, Verona, Italy), according to a standard imaging protocol [15]. In the Netherlands, CBCT scans of the patients were made at the Department of Oral Radiology at the Academic Centre for Dentistry Amsterdam (ACTA). In China, CBCT scans of the patients were made at the Department of Oral Radiology, School of Dentistry, Shandong University.

During the imaging procedure, automatic exposure control was applied, and the patients were positioned in a supine position with the Frankfort horizontal (FH) plane being perpendicular to the floor [15]. They were instructed to maintain light contact between the molars in natural occlusion, to keep quiet breathing, and to avoid swallowing and other movements during the scanning period. The exposure settings were 110 kV, 4 mA, 0.3 mm voxel size, 3.6 s exposure time (pulsed radiation), and 18–36 s scanning time, depending on the size of the patient [15]. To get a standardized head position of each CBCT image, re-orientation was performed by adjusting the palatal plane (the plane crossing anterior nasal spine (ANS)-posterior nasal spine (PNS)) being parallel to the global horizontal plane in the sagittal view, and perpendicular to the global horizontal plane in the axial view [20]. For further analysis, the images were saved as digital imaging and communications in medicine (DICOM) files. All images were presented to the observers in a room with dimmed light.

### Primary outcome variable: anatomical balance of the upper airway

**Fig. 1:**
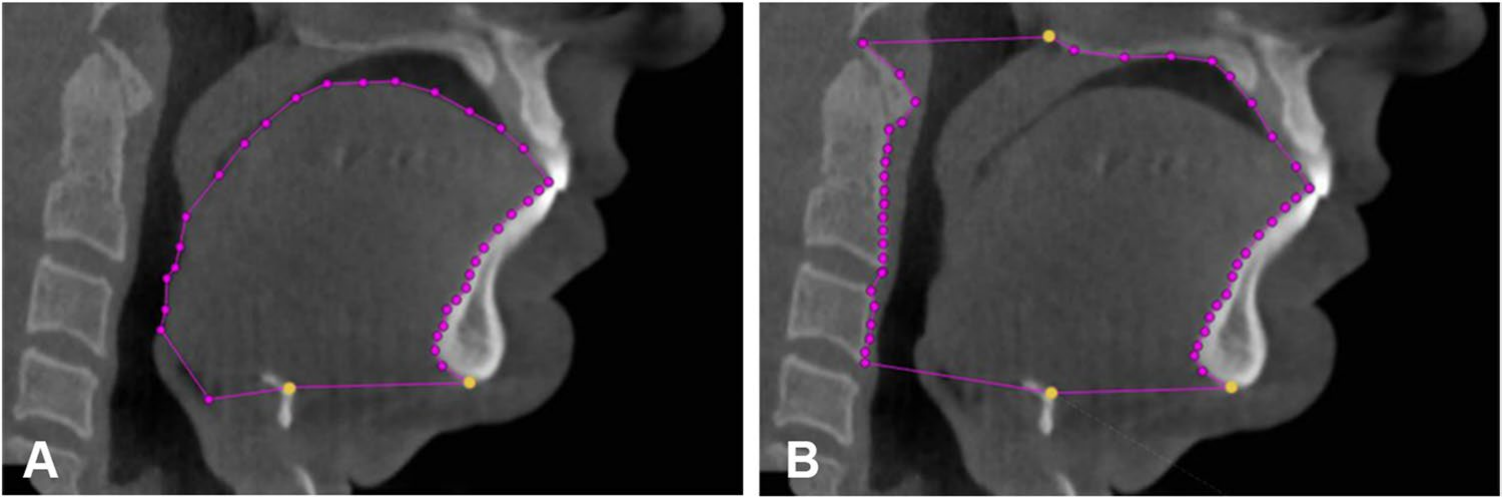
The measurements of the tongue area and the maxillomandibular enclosure area on the mid-sagittal plane of a CBCT image (an example from the Dutch group). **A** The tongue area. **B** The maxillomandibular enclosure area

Anatomical balance of the upper airway was calculated using the ratio of the tongue area and the maxillomandibular enclosure area, which were measured on the mid-sagittal plane of CBCT imaging using 3Diagnosys® software (v5.3.1, 3diemme, Cantu, Italy). The tongue area (mm^2^) was determined by the area enclosed by the point hyoid, menton, the contour of the frontal teeth and the tongue, and the base of the epiglottis (Fig. 1A). The maxillomandibular enclosure area (mm^2^) was determined by the area enclosed by the point hyoid, menton, the contour of the front teeth, the hard palate, the posterior nasal spine, and the anterior boundary of the second and third cervical vertebra (Fig. 1B).

The values of the anatomical balance range between 0 and 1, and a larger value of the anatomical balance means the tongue occupied a larger section of the maxillomandibular enclosure area compared to a smaller value.

### Secondary outcome variables

**Fig. 2:**
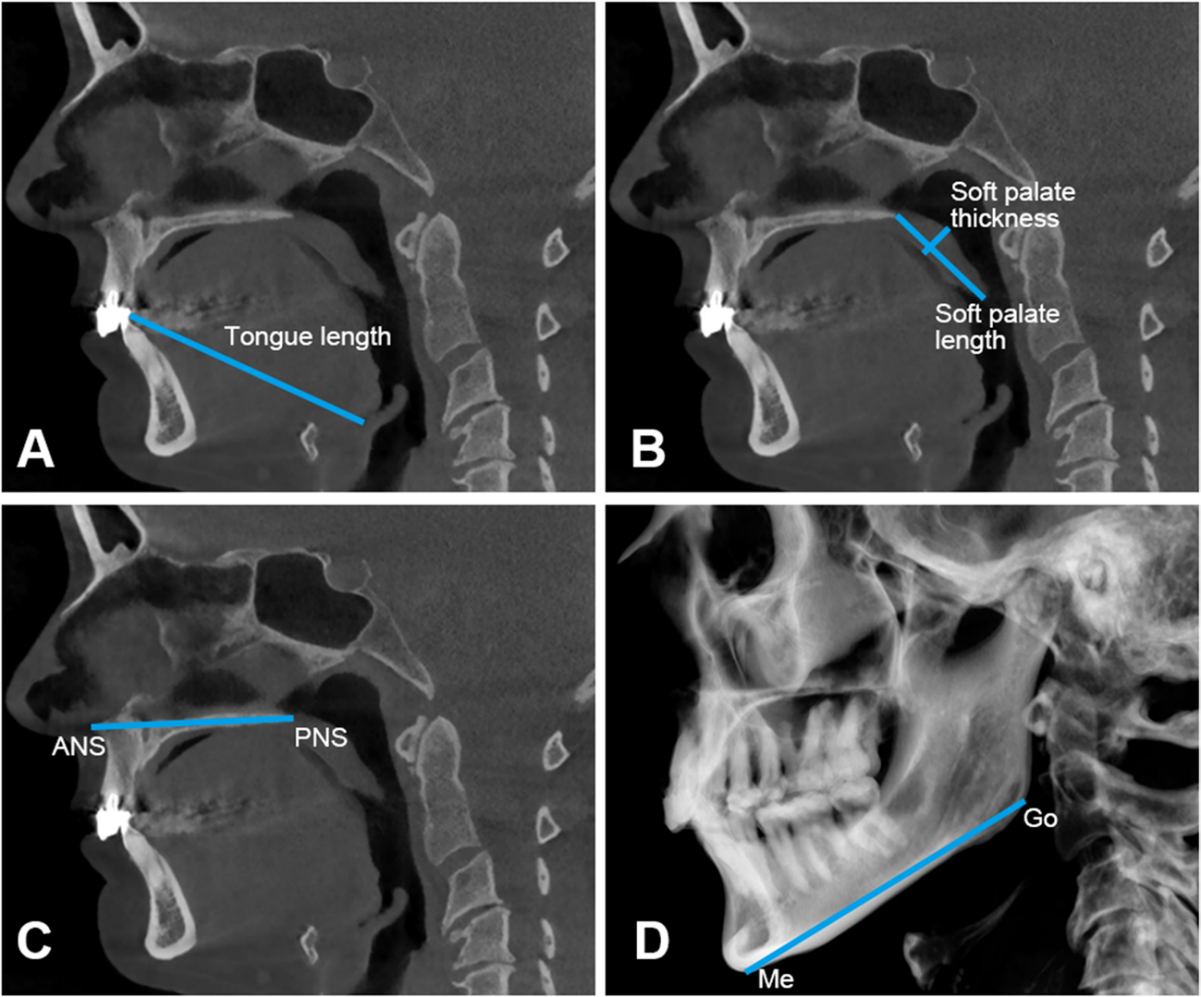
Illustration of the secondary outcome variables of the upper airway morphology (an example from the Dutch group). **A** Tongue length. **B** Soft palate length and soft palate thickness. **C** Maxilla length: distance between ANS (anterior nasal spine) and PNS (posterior nasal spine). **D** Mandibular length: distance between Go (gonion) and Me (menton)

Secondary outcome variables of the upper airway morphology, including the structures of maxillomandibular enclosure, tongue, soft palate, the maxilla, and the mandible, were measured using 3Diagnosys® software (v5.3.1, 3diemme, Cantu, Italy). The definitions and illustrations of the secondary variables are shown in Table 1 and Fig. 2.

**Table 1:** Definitions of the primary and secondary outcome variables of the upper airway morphology

Structures	Variables	Definitions
Primary outcome variable		
Anatomical balance	Anatomical balance	Ratio of the tongue area and the maxillomandibular enclosure area
Secondary outcome variables		
Maxillomandibular enclosure	Maxillomandibular enclosure area	⁠Area enclosed by the point hyoid, menton, the contour of the front teeth, the hard palate, the posterior nasal spine, and the anterior boundary of the second and third cervical vertebra.
Tongue	Tongue area	Area enclosed by the point hyoid, menton, the contour of the frontal teeth and the tongue, and the base of the epiglottis.
	Tongue length	Distance between the tip of tongue and the base of epiglottis.
Soft palate	Soft palate length	Distance between the posterior nasal spine (PNS) and tip of soft palate.
	Soft palate thickness	Maximum thickness of soft palate measured on the line perpendicular to the line of PNS—tip of soft palate.
Maxilla	Maxilla length	Distance between the anterior nasal spine (ANS) and PNS.
Mandible	Mandibular length	Distance between the gonion (Go) and menton (Me).

### Statistical analysis

The Shapiro-Wilk test was used to test whether the data were normally distributed. The Mann-Whitney *U* test (for non-normally distributed variables), the independent *t*-test (for normally distributed variables), and the chi-squared test (for categorical variables) were used to compare the demographic characteristics, and the primary and secondary outcome variables of the upper airway morphology between the Dutch and Chinese OSA groups. Bonferroni-Holm correction was applied to the comparisons of secondary outcome variables of the upper airway [21]. Statistical analyses were performed using IBM® SPSS® Statistics for Macintosh, Version 26 (IBM Corp., Armonk, NY, USA).

The effect size was calculated for the anatomical balance by the software G*power (version 3.1.9, Franz Faul, Universität Kiel, Germany).

## Results

### Baseline characteristics

There were 28 Dutch patients and 24 Chinese patients fulfilling the requirements of inclusion and exclusion criteria included in the study. The demographic characteristics of the Dutch and Chinese patients are shown in Table 2. There were no significant differences in age, sex, body mass index (BMI), and apnea-hypopnea index (AHI) between the Dutch and Chinese patients (*P* = 0.14–0.76).

**Table 2:** Baseline demographic characteristics of the Dutch and Chinese OSA patients included in the study

	Dutch patients (*n* = 28)	Chinese patients (*n* = 24)	*T* or *X*^2^	*P*
Age (year)	46.6 ± 14.1	41.0 ± 12.4	1.50 (*T*)	0.14
Sex (female vs male)	8 vs 20	4 vs 20	1.03 (*X*^2^)	0.31
BMI (kg/m^2^)	26.8 ± 3.5	26.5 ± 3.3	0.31 (*T*)	0.76
AHI (events/hour)	15.7 ± 7.1	16.5 ± 7.8	−0.36 (*T*)	0.72

Data are shown as mean ± standard deviation (SD); *T* independent *t*-test, *X*^2^ chi-squared test; *BMI* body mass index, *AHI* apnea-hypopnea index

### Comparisons of the upper airway morphology

The variables of the upper airway morphology of the Dutch and Chinese patients are shown in Table 3. The Dutch group had a significantly larger tongue area (*T* = 2.60, *P* = 0.01) and a larger tongue length (*Z* = −3.14, *P* < 0.01) compared to the Chinese group. On the other hand, the Chinese group had a smaller maxilla length compared to the Dutch group (*T* = 7.34, *P* < 0.01). However, the anatomical balance of the upper airway of both groups was not significantly different (*T* = 1.43, *P* = 0.16). The effect size d, which is defined as a standardized difference in the means of the anatomical balance of the upper airway between both groups, was 0.4, which can be regarded as between small and medium (the effect size d of 0.20 is small, one of 0.5 is medium, and one of 0.8 is large [22]).

**Table 3:** The variables of the upper airway morphology of the Dutch and Chinese patients

Variables	Dutch patients (*n* = 28)	Chinese patients (*n* = 24)	*T*/*Z*	*P*
Primary outcome variable				
Anatomical balance	0.70 ± 0.1	0.68 ± 0.0	1.43 (*T*)	0.16
Secondary outcome variables				
*Maxillomandibular enclosure size*				
Maxillomandibular enclosure area (cm^2^)	4.9 ± 0.6	4.7 ± 0.4	1.81 (*T*)	0.08
*Tongue*				
Tongue area (cm^2^)	3.4 ± 0.4	3.2 ± 0.3	2.60 (*T*)	0.01*
Tongue length (cm)	7.6 (7.0, 8.1)	6.9 (6.6, 7.4)	-3.14 (*Z*)	< 0.01*
*Soft palate*				
Soft palate length (cm)	4.1 (3.8, 4.4)	4.0 (3.7, 4.1)	-1.65 (*Z*)	0.10
Soft palate thickness (cm)	1.0 ± 0.2	0.9 ± 0.2	2.17 (*T*)	0.04
*Maxilla*				
Maxilla length (cm)	5.6 ± 0.4	4.9 ± 0.3	7.34 (*T*)	< 0.01*
*Mandible*				
Mandibular length (cm)	7.3 ± 0.6	7.1 ± 0.5	1.55 (*T*)	0.13

Normally distributed data are shown as mean ± standard deviation (SD); non-normally distributed data are shown as median (25th percentile, 75th percentile); *T* independent *t*-test, *Z* Mann-Whitney *U* test; *Significant difference with Bonferroni-Holm correction

## Discussion

The aim of the present study was to compare the anatomical balance of the upper airway between Dutch and Chinese patients with OSA. The results indicated that the Dutch group had a significantly larger tongue area and a larger tongue length compared to the Chinese group, while the Chinese group had a smaller maxilla length compared to the Dutch group. However, the anatomical balance of the upper airway of both groups was not significantly different.

### Comparisons of the upper airway morphology

For the primary outcome variable, we did not find a significant difference in the anatomical balance of the upper airway between Dutch and Chinese patients with OSA. Further, the observed effect size d for the difference in the anatomical balance of the upper airway was 0.4, which is between small and medium. With this effect size, the difference in the anatomical balance between both groups may be not clinically relevant either [23, 24]. In contrast to our results, a study of Schorr et al. [11] has suggested that Caucasians with OSA have a larger anatomical imbalance compared with the Japanese-Brazilians with OSA. However, for calculating the anatomical balance, they used the volume of the bony tissue rather than the volume of the bony enclosure as the denominator, which may cause bias and explain the different results as compared to our results. A study of Lee et al. [10] has suggested a similar anatomical balance between Caucasian and Chinese OSA groups, which is similar to our results. However, they used a simplified definition of the anatomical balance, defined as ratios of BMI to mandibular and maxillary bony dimensions, which may be less accurate. The definition used in the present study has been used widely in the literature to investigate the role of the anatomical balance of the upper airway in the pathogenesis and treatment of OSA [13, 14, 25]. Thus, by using a more generalized and accurate definition, the present study confirms that the anatomical balance of the upper airway is similar in Dutch and Chinese OSA groups.

For the secondary outcome variables, the Dutch group had a significantly larger tongue size and larger tongue length compared to the Chinese group. These results are similar to those of previous studies [9-12], which indicates that when the OSA severity is similar, Caucasian patients are more overweight, while Asian patients tend to have a smaller maxilla and mandible.

Previous studies have suggested that the craniofacial skeletal difference between Asians and Caucasians, such as restricted bony structures in Asians, is an important reason for a greater tendency of OSA development in Asians [9, 26]. However, both bony structures and soft tissues can influence the upper airway morphology. By taking into account both factors, the present study indicates that the anatomical imbalance may be similar for both groups. However, in addition to the anatomical factor, several non-anatomical factors are also crucial determinants for upper airway collapse, such as impaired upper airway dilator muscle activity, ventilatory control stability (i.e., high loop gain), and low arousal threshold [27]. The study of Lee et al. [28] has suggested that a low arousal threshold is a less common mechanism in the pathogenesis of the Chinese OSA group compared to the Caucasian OSA group. Further, the study of O’Driscoll et al. [29] has suggested that the loop gain is significantly higher in the Caucasian group than in the Chinese group. However, both studies included moderate-to-severe OSA patients, which may represent a different study sample as compared to our study. To the best of our knowledge, the difference in the non-anatomical factors between both races in patients with mild-to-moderate OSA is not clear yet. Understanding the individual pathogenesis can help in a personalized treatment approach in OSA [27]. Therefore, future research is needed to investigate the roles of anatomical and non-anatomical factors in the pathogenesis of OSA.

### Demographic characteristics

Lee et al. [10] have suggested that the referral approach for the clinical assessment of OSA may be influenced by the differences in socioeconomic status, cultural, and environmental factors between the Caucasian and Chinese groups. This is consistent with the phenomenon that we discovered during the recruitment process. There were more patients with severe OSA referred to the sleep laboratory in China than in the Netherlands. To minimize the selection bias, we only recruited patients with mild to moderate OSA in both groups. Besides, both groups were similar in BMI, age, and sex. Therefore, the comparisons of the upper airway morphology between both groups were not biased by these factors.

### Clinical relevance

Based on the non-significant results of the present study, it is possible that the anatomical balance of the upper airway plays a similar role in the pathogenesis of OSA in both races. Therefore, it may be speculated that treatment, which mainly targets the anatomical factors, might result in similar treatment results in both groups. Further studies will be performed in our lab to evaluate the treatment effects of therapy in both races.

## Limitations

There are several limitations in the present study. First, this study is a multi-center study recruiting Dutch and Chinese patients separately from two sites, which might cause selection bias. However, one of the investigators (H.C.) visited both clinics to make sure that the protocol was implemented in the same way in both the Netherlands and China. Second, in our study, we defined the Dutch and Chinese races of the patients based on their family trees and names, which may cause selection bias. However, the definition of race in the medical literature is not always clear [30]. Contrary to other studies, our definition of race relied on family history rather than on the assumptions of the investigators, which could be a better approach in epidemiological studies [10]. Third, there are 56 ethnic groups in China, and previous studies concluded that the genetic structure is different among different ethnic groups in China [31, 32]. As the craniofacial structure of patients is influenced by genetic factors [33], it is possible that there could be a difference in the craniofacial structure from different ethnic groups in China. In this study, we therefore recruited the Chinese patients only from the Han ethnic group. We excluded the effect of the living environment by recruiting only Dutch patients living in the Netherlands and Chinese patients living in Shandong, China. The measurements of the tongue area and the maxillomandibular enclosure area were based on the mid-sagittal plane of the CBCT imaging, and not on 3D volume measurements. The volume measurements in CBCT images were limited by the difficulty in discriminating between the different soft-tissue structures (due to similar Hounsfield units).

## Conclusion

Within the limitations of this study, we conclude that there is no significant difference in the anatomical balance of the upper airway between Dutch and Chinese patients with mild to moderate OSA.

## Data Availability

The datasets generated during and/or analyzed during the current study are available from the corresponding author on reasonable request.
